# Influence of surgery involving tendons around the knee joint on ankle motion during gait in patients with cerebral palsy

**DOI:** 10.1186/s12891-018-2003-0

**Published:** 2018-03-15

**Authors:** Seung Yeol Lee, Soon-Sun Kwon, Chin Youb Chung, Kyoung Min Lee, Ki Hyuk Sung, Sangwoo Kim, Moon Seok Park

**Affiliations:** 1 Department of Orthopaedic Surgery, Ewha Womans University Mokdong Hospital, Seoul, South Korea; 2 Department of Mathematics, College of Natural Science, Ajou University, Suwon, Gyeonggi South Korea; 3 Department of Orthopaedic Surgery, Seoul National University Bundang Hospital, 300 Gumi-Dong, Bundang-Gu, Seoungnam, Gyeonggi 463-707 South Korea; 4 Department of Orthopaedic Surgery, Seoul National University College of Medicine, Seoul, South Korea

**Keywords:** Cerebral palsy, Gait analysis, Ankle kinematics, Distal hamstring lengthening, Rectus femoris transfer

## Abstract

**Background:**

Simultaneous motion of the knee and ankle joints is required for many activities including gait. We aimed to evaluate the influence of surgery involving tendons around the knee on ankle motion during gait in the sagittal plane in cerebral palsy patients.

**Methods:**

We included data from 55 limbs in 34 patients with spastic cerebral palsy. Patients were followed up after undergoing only distal hamstring lengthening with or without additional rectus femoris transfer. The patients’ mean age at the time of knee surgery was 11.2 ± 4.7 years, and the mean follow-up duration was 2.2 ± 1.5 years (range, 0.9–6.0 years). Pre- and postoperative kinematic variables that were extracted from three-dimensional gait analyses were then compared to assess changes in ankle motion after knee surgery. Outcome measures included ankle dorsiflexion at initial contact, peak ankle dorsiflexion during stance, peak ankle dorsiflexion during swing, and dynamic range of motion of the ankle. Various sagittal plane knee kinematics were also measured and used to predict ankle kinematics. A linear mixed model was constructed to estimate changes in ankle motion after adjusting for multiple factors.

**Results:**

Improvement in total range of motion of the knee resulted in improved motion of the ankle joint. We estimated that after knee surgery, ankle dorsiflexion at initial contact, peak ankle dorsiflexion during stance, peak ankle dorsiflexion during swing, and dynamic range of motion of the ankle decreased, respectively, by 0.4° (*p* = 0.016), 0.6° (*p* < 0.001), 0.2° (*p* = 0.038), and 0.5° (*p* = 0.006) per degree increase in total range of motion of the knee after either knee surgery. Furthermore, dynamic range of motion of the ankle increased by 0.4° per degree increase in postoperative peak knee flexion during swing.

**Conclusions:**

Improvement in total knee range of motion was found to be correlated with improvement in ankle kinematics after surgery involving tendons around the knee. As motion of the knee and ankle joints is cross-linked, surgeons should be aware of potential changes in the ankle joint after knee surgery.

**Electronic supplementary material:**

The online version of this article (10.1186/s12891-018-2003-0) contains supplementary material, which is available to authorized users.

## Background

Stiff-knee gait and crouch gait are among the most common gait problems in ambulatory patients with cerebral palsy (CP) [1, 2]. Stiff-knee gait results in limited flexion and extension of the knee, and a restricted arc of motion during the swing phase [3]. Crouch gait, a major sagittal plane deviation, is defined as excessive ankle dorsiflexion combined with knee and hip flexion during the stance phase [4]. To optimize knee function in CP patients with abnormal gait, various surgical treatments, such as rectus femoris transfer (RFT) for stiff knee gait, and distal hamstring lengthening (DHL) and distal femoral osteotomy for flexed knee gait, can be considered as part of single-event multilevel surgery (SEMLS).

Many patients with CP also undergo tendo-Achilles lengthening as part of SEMLS to release the tightness preventing suitable ankle dorsiflexion. However, some patients with stiff- or flexed-knee gait show a relatively good ankle range of motion (ROM) [5, 6]. Thus, knee surgery procedures such as RFT or DHL without additional ankle surgery can be considered in these patients. Cross-linkage of the knee and ankle; however, still needs to be considered. Specifically, the gastrocnemius, a biarticular muscle that crosses the knee and ankle, acts as both a knee and ankle plantar flexor. The resultant simultaneous motion of the knee and ankle joints is required for many activities including standing, running, swimming, and cycling [7]. In addition, adequate coupling of plantar flexion and knee extension [8] regulates the direction and modulus of ground reaction force, which subsequently acts to optimize gait.

Given this relationship between the knee and ankle, we hypothesize that changes in the kinematics of the knee after knee surgery affect ankle motion during walking in patients with CP. For example, DHL- or DHL with RFT-induced improvement in knee motion during the swing phase may result in improved heel strike at initial contact in the gait cycle, which subsequently affects ankle motion during the stance phase. Indeed, many previous studies have reported improved ankle kinematics after knee surgeries in patients with CP, though these studies included patients who underwent concurrent surgeries that may have affected ankle kinematics [9–12]. In addition, changes in ankle motion during gait have not, to the best of our knowledge, been described in patients with CP who have undergone DHL or DHL with RFT specifically. Therefore, the present study aimed to evaluate the extent to which surgery involving tendons around the knee joint influences ankle motion during gait in the sagittal plane in patients with CP.

## Methods

### Ethical statement

This study was approved by the institutional board of our hospital, a tertiary referral center for CP. Informed consent was waived because of the retrospective nature of this study.

### Patient recruitment

We reviewed the medical records of patients with spastic CP who were followed up after RFT or DHL, and who had undergone pre- and postoperative three-dimensional (3D) gait analysis between January 1995 and December 2015. Patients were included in this study if they did not meet the indications for tendo-Achilles lengthening or gastrocnemius recession (these indications are: the ankle is not correctable to more than a neutral position, as measured during knee extension; and increased plantar flexion in the stance phase of walking). Exclusion criteria were as follows: gross motor function classification system (GMFCS) level IV or V, any previous or concurrent surgeries as a part of SEMLS, and incomplete or missing 3D gait analysis data. Age at the time of surgery, sex, anatomic type of CP, GMFCS level, and details of concomitant surgical procedures were obtained from patient medical records.

### Operation protocol

RFT and DHL were performed by 2 orthopedic surgeons who had 27 and 11 years of experience in orthopedics. Both surgeons followed the same treatment approach. Indications for RFT were as follows: positive Duncan Ely test, knee flexion angle ≥15° less than the total range during the swing phase, and delayed peak knee flexion during the swing phase [13, 14]. When RFT was performed without DHL, the rectus femoris was transferred to the sartorius. In cases where RFT and DHL were performed concomitantly (DHT with RFT group), the gracilis tendon was used as the transfer site. Some patients also underwent DHL only (DHL only group). DHL was performed in patients who exhibited an increased popliteal angle and increased knee flexion at initial contact, as verified by preoperative 3D gait analysis. The procedure included gracilis lengthening, semitendinosus transfer to the adductor magnus, and aponeurotic lengthening of the semimembranosus.

### Acquisition of kinematic data

3D gait analysis was performed pre- and postoperatively using a Vicon 370 motion capture system (Oxford Metrix, Oxford, UK) equipped with 7 cameras and 2 force plates (Advanced Medical Technology Incorporation, MA, USA). The Vicon Plug-in Gait marker model was used to place the markers. To obtain kinematic data, patients were recorded walking barefoot along a 9-m walkway 3 times, with an interval of approximately 30 s between each recording. The 3 trials were then averaged to determine the index variable values [13]. Next, pre- and postoperative kinematic variables were compared to assess changes in ankle motion after surgery involving tendons around the knee. Specifically, kinematic variables including knee flexion at initial contact, minimum knee flexion in the stance phase, peak knee flexion in the swing phase, total knee ROM, and knee flexion in the terminal swing phase were used to assess knee kinematics after DHL and RFT. On the other hand, ankle dorsiflexion at initial contact, peak ankle dorsiflexion in the stance phase, peak ankle dorsiflexion in the swing phase, and dynamic ROM were used as outcome measures for ankle motion changes after surgery.

### Statistical analysis

The Kolmogorov-Smirnov test was used to verify the normal distribution of continuous variables, which were described using means, standard deviations, and frequencies. Changes in each gait parameter after surgery were analyzed using paired-t tests. A linear mixed model (LMM) was then constructed to estimate changes in ankle motion between pre- and postoperative evaluations, with adjustment for multiple factors including sex and knee surgery type (i.e., DHL only or DHL with RFT). After univariate analysis, changes in the kinematics of the knee were considered fixed effects, whereas the interval between the 3D gait analyses, laterality (i.e., right or left), age, and each subject were considered random effects (Additional file 1).

Four LMMs were constructed for each ankle kinematic variable of interest (i.e., ankle dorsiflexion at initial contact, peak ankle dorsiflexion in the stance phase, peak ankle dorsiflexion in the swing phase, and dynamic ankle ROM), and the variance components covariance structure was used. The restricted maximum likelihood estimation method was used to produce an unbiased estimator. The adequacy of the models was determined using the Akaike information criterion (AIC) and Bayesian information criterion (BIC), with smaller values preferred for model selection. All models had low AIC/BIC scores (872.9/870.9 for peak ankle dorsiflexion at initial contact, 850.5/848.5 for peak ankle dorsiflexion during stance, 820.7/818.7 for peak ankle dorsiflexion during swing, and 883.0/881.0 for dynamic ankle ROM), and were thus accepted as valid representations of kinematic measurements.

Data were analyzed using SAS version 9.4 (SAS Institute, Cary, NC). All statistical tests were two-tailed. Confidence intervals (CIs) were considered significant when they did not include zero, and *p*-values < 0.05 were considered significant.

## Results

**Fig. 1:**
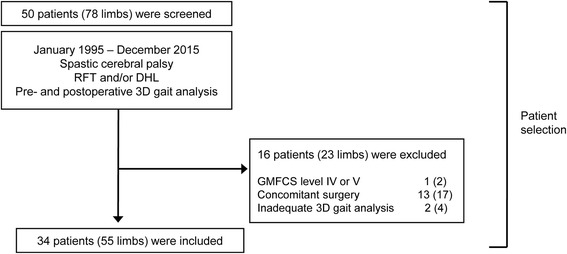
Inclusion and exclusion criteria. *RFT* rectus femoris transfer; *DHL* distal hamstring lengthening, *GMFCS* gross motor function classification system

Seventy-eight lower limbs from 50 patients met the inclusion criteria. After applying the exclusion criteria, a total of 55 limbs from 34 patients remained for inclusion in this study (Fig. 1). The patients’ mean age at the time of knee surgery was 11.2 ± 4.7 years, and postoperative 3D gait analysis was performed at a mean of 0.9 ± 1.3 years after surgery (Table 1). The mean follow-up duration was 2.2 ± 1.5 years (range, 0.9–6.0 years). Five patients (9 limbs) underwent postoperative 3D gait analysis twice, while 29 patients (46 limbs) underwent the examination once.

**Table 1:** Patient demographics

Parameter	Value	
No. of patients (M / F)	34 (18/16)	
No. of limbs (M / F)	55 (29/26)	
Laterality (R / L)	27/28	
Anatomical type (hemi−/di−/quadriplegia)	27/2/26	
GMFCS level (patients /limbs)	I	13 (19)
	II	15 (25)
	III	6 (11)
Mean age at surgery (years)	11.2 (SD 4.7)	
Knee surgery (DHL / RFT/ both) (limbs)	27 / 2 / 26	
Interval between pre- and first postoperative 3D gait analysis (years)	0.9 ± 1.3	
Mean follow-up duration (years)	2.2 ± 1.5 (range, 0.9 to 6.0)	

*M* male, *F* female, *R* right, *L* left, *DHL* distal hamstring lengthening, *RFT* rectus femoris transfer

All knee kinematic variables improved between pre- and postoperative 3D gait analysis (Table 2). The mean total knee ROM increased from 38.9° to 50.2° overall (specifically, from 39.5° to 47.8° in the DHL group and from 38.8° to 52.2° in the DHL with RFT group) after surgery (*p* < 0.001) (Table 2) [14, 15].

**Table 2:** Changes in gait parameters after surgery involving tendons around the knee

	Kinematics	^a^Reference value in children [15, 16]	Preoperative value(°)	Postoperative value (°)	Changes in parameter after the surgery (°)		
					Mean value	CI	*P* value
Knee	Knee flexion at IC	3.4–14.4	31.9 (SD 12.7)	22.8 (SD 9.7)	−9.1 (SD 14.1)	−12.9 – −5.3	< 0.001
	Peak knee flexion during swing	55.6–61.6	55.4 (SD 14.3)	58.3 (SD 11.0)	2.9 (SD 16.1)	−1.5 – 7.2	0.194
	Total knee ROM	46.8–57.2	38.9 (SD 14.0)	50.2 (SD 11.4)	11.2 (SD 14.6)	7.3–15.2	< 0.001
	Minimum knee flexion in the stance	7.6	16.5 (SD 14.9)	5.1 (SD 1.5)	−8.4 (SD 16.8)	−12.9 – −3.8	0.001
Ankle	Ankle dorsiflexion at IC	4.2	4.8 (SD 19.8)	3.5 (SD 8.7)	−1.2 (SD 19.5)	−6.5 – 4.0	0.641
	Peak ankle dorsiflexion during stance	9.0–14.8	21.6 (SD 21.2)	18.6 (SD 8.2)	−3.0 (SD 20.3)	−8.5 – 2.5	0.277
	Peak ankle dorsiflexion during swing	2.1–5.6	17.2 (SD 9.8)	15.2 (SD 7.3)	−2.0 (SD 10.2)	−4.8 – 0.7	0.142
	Dynamic ROM of the ankle	26.6–31.7	14.9 (SD 25.2)	11.5 (SD 7.5)	−3.4 (SD 22.7)	−9.6 – 2.7	0.269

^a^Reference value in children is the range of mean values of previous studies

**Fig. 2:**
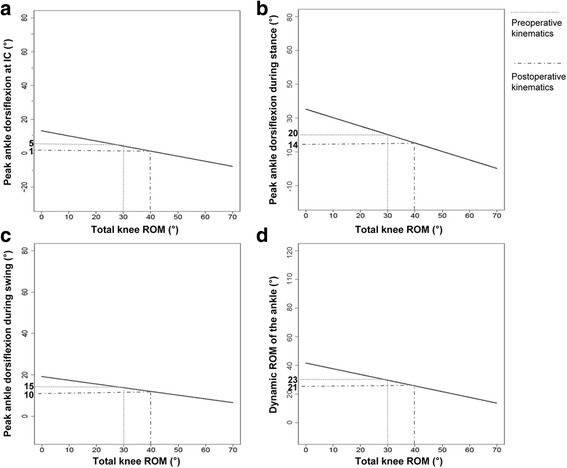
Linear mixed model describing changes in ankle kinematics associated with postoperative improvement in total knee range of motion (ROM). The sloping lines indicate estimated changes in ankle kinematics according to the improvement of total knee ROM. For instance, one patient demonstrated 30° total knee ROM, 5° ankle dorsiflexion at initial contact (**a**), 20° peak ankle dorsiflexion in the stance phase (**b**), 15° peak ankle dorsiflexion in the swing phase (**c**), and 23° dynamic ROM of the ankle (**d**). If total knee ROM increased to 40° after the knee surgery, estimated ankle dorsiflexion at initial contact, peak ankle dorsiflexion in the stance and swing phases, and dynamic ankle ROM will be 1° (**a**), 14° (**b**), 10° (**c**), and 21° (**d**)

LMM analysis showed that ankle kinematics were influenced by improvement in knee kinematics (Fig. 2), with improvement in total knee ROM resulting in decreased motion of the ankle joint. We estimated that ankle dorsiflexion at initial contact, peak ankle dorsiflexion in the stance phase, peak ankle dorsiflexion in the swing phase, and dynamic ankle ROM decreased by 0.4° (*p* = 0.016), 0.6° (*p* < 0.001), 0.5° (*p* = 0.038), and 0.2° (*p* = 0.006), respectively, per degree increase in total knee ROM after surgery (Table 3). Furthermore, dynamic ankle ROM increased by 0.4° per degree increase in postoperative peak knee flexion in the swing phase. We also found that pre- and postoperative ankle dorsiflexion at initial contact, peak ankle dorsiflexion in stance, and dynamic ankle ROM were smaller in the DHL with RFT group than in the DHL only group (Table 3). However, age at the time of knee surgery did not significantly affect ankle kinematics.

**Table 3:** Linear mixed model describing estimated and fixed effects on ankle kinematics after surgery involving tendons around the knee

Effect	Peak ankle dorsiflexion at IC			Peak ankle dorsiflexion during stance			Peak ankle dorsiflexion during swing			Dynamic ankle ROM		
	Estimate	CI	*P*-value	Estimate	CI	*P* -value	Estimate	CI	*P* -value	Estimate	CI	*P* -value
Intercept	13.8	−2.4– 30.1	0.091	25.6	8.4–42.7	0.005	8.0	−1.4–17.5	0.094	31.7	11.8–51.6	0.003
Age	−0.3	−0.9 – 0.3	0.314	−0.1	−0.7 – 0.5	0.749	− 0.3	− 0.6 – 0.1	0.119	− 0.2	− 0.9 – 0.5	0.583
Laterality (Right)	1.5	−4.0 – 6.9	0.594	1.2	−4.5 – 7.0	0.669	−1.0	−4.2 – 2.1	0.519	0.6	−6.1 – 7.3	0.862
DHL only	6.2	0.5–11.8	0.032	6.8	0.9–12.7	0.025	−1.0	−4.3 – 2.3	0.540	8.2	1.3–15.1	0.020
^a^Knee flexion at IC	0.2	−0.6 – 1.1	0.603	−0.5	−1.4 – 0.4	0.287	−0.7	−1.8 – 0.3	0.179	−0.4	−0.9 – 0.1	0.152
^a^Peak knee flexion during swing	0.0	−0.4 – 0.4	0.906	0.3	−0.1 – 0.7	0.132	0.0	−0.5 – 0.5	0.952	0.4	0.2–0.6	< 0.001
^a^Total knee ROM	−0.4	− 0.7 – − 0.1	0.016	−0.6	−1.0 – − 0.3	< 0.001	−0.5	− 1.0 – − 0.2	0.006	-0.2	−0.4 – 0.0	0.038
^a^Knee flexion in terminal swing	−0.2	−1.0 – 0.6	0.618	0.3	−0.6 – 1.1	0.515	0.6	−0.4 – 1.5	0.230	0.2	−0.2 – 0.7	0.289

*CI* 95% confidence interval, *IC* initial contact, *ROM* range of motion

^a^Each degree of increase in kinematics

## Discussion

Our study is the first to describe how surgery involving the tendons around the knee joint affects ankle motion during gait in patients with CP [16]. We have shown that DHL- or DHL with RFT-induced improvement in total knee ROM results in an improvement in ankle kinematics in the sagittal plane during gait.

Most sports and activities of daily living require the concomitant movement of various joints. The coordination of this joint movement is essential in human gait, which is the result of a combination of forces that allow maximum efficiency to be achieved at minimum cost [17]. The gastrocnemius muscle plays a role in this coordination, crossing the knee and ankle to act as both a knee flexor and an ankle plantar flexor in the sagittal plane. The resultant coupling of plantar flexion and knee extension is thought to underlie the knee and ankle kinematics that regulate the direction and modulus of ground reaction force to optimize gait [8]. Therefore, physicians should be aware of potential changes in ankle kinematics after knee surgery.

The present study found that improvement in total knee ROM resulted in decreased (i.e. improved) motion of the ankle joint in terms of peak ankle dorsiflexion at initial contact and during the stance and swing phases, as well as dynamic ankle ROM. Specifically, we estimated that peak ankle dorsiflexion at initial contact, peak ankle dorsiflexion in the stance phase, peak ankle dorsiflexion in the swing phase, and dynamic ankle ROM decreased by 0.4°, 0.6°, 0.2°, and 0.5°, respectively. Although these improvements seem small, they must be considered in terms of degree increase in total knee ROM. Because the ankle ROM is smaller than that of the knee during walking [15], these improvements in ankle kinematics actually represent substantial changes.

Furthermore, although these findings do not intuitively represent an improvement, they should be understood in the context of our patients undergoing surgery for tightness or shortness in the hamstring or rectus femoris. As such, the average preoperative total knee ROM was lower in our patients than in healthy children of a similar age (our patients, 38.9° vs. healthy children, 46.8°-57.2°), as was dynamic ankle ROM during walking [15, 18]. In contrast, peak ankle dorsiflexion parameters during walking were larger in our cohort than in healthy children, which might be related to weakness of the triceps surae. However, we found that improvement in knee kinematics after knee surgery was associated with a decrease in peak ankle dorsiflexion during gait. This suggests that increased preoperative ankle dorsiflexion represents a means of secondary compensation, triceps surae weakness, for decreased knee ROM. This is consistent with what is known about loss of knee ROM. Such loss of knee function is associated with gait problems including foot clearance and heel strike [13], which can be compensated for by increasing ankle dorsiflexion through the gait cycle. Thus, the decreased ankle dorsiflexion we found after surgery indicates less need for compensation and improved function. In addition, peak knee flexion during the swing phase showed a positive correlation with dynamic ankle ROM, but no correlation with ankle dorsiflexion at initial contact and during the stance and swing phases. We therefore suggest that improvement in peak knee flexion might allow effective push-off of the foot during the initial swing phase.

We also found that postoperative dynamic ROM of ankle and as well as peak ankle dorsiflexion at initial contact and in the stance phase, were larger in the DHL only group than in the DHL with RFT group. Since total knee ROM was negatively correlated with peak ankle dorsiflexion and dynamic ankle ROM during walking in our study, patients who underwent DHL with RFT also had larger improvements in total knee ROM than those who underwent only DHL. This is indicative of poorer results in the DHL only group in terms of reducing the need for secondary compensation and enabling a larger decrease in ankle kinematics during gait to be achieved. This is unsurprising given that patients with stiff-knee gait generally required both DHL and RFT, the latter of which has been suggested to restore knee motion during the swing phase [3, 13]. Another possible reason for greater dynamic ankle ROM in the DHL only group involves patient selection. Patients who underwent DHL only exhibited hamstring tightness and shortness, which can contribute to jump-knee gait or crouch gait [19–21]. Since patients with jump-knee gait who required surgery for heel cord tightness (not hamstring tightness) were excluded, characteristics specific to patients with crouch gait might have contributed to the increased dynamic ankle ROM and as well as the increased ankle dorsiflexion at initial contact and in the stance phase, seen in the DHL only group.

Regarding other disadvantages of DHL, there are concerns that DHL may aggravate anterior pelvic tilt and lumbar lordosis, as well as induce crouch gait [11, 23, 24], despite its effectiveness at reducing knee flexion and improving knee motion [25]. Recent studies have also found that preservation of hip extension power and hip extension are better with than without the hamstring transfer procedure [5, 12, 26]. In our study, total knee ROM improved after knee surgery, and was correlated with a decrease in peak ankle dorsiflexion and dynamic ankle ROM during gait. These results suggest that in patients with crouch gait, excessive dorsiflexion of the ankle during gait may improve after DHL. Thus, we believe that in patients with CP, the effect of DHL on ankle motion might lead to an overall improvement in gait.

Despite the insights provided by this study, there are some limitations that should be addressed. First, because of the retrospective nature of this study, it was not possible to ensure consistency in the timeline of our evaluations. Therefore, the interval between pre- and postoperative 3D gait analyses varied with each case considered. To overcome the heterogeneity of this data set and adjust the interval between examinations, we used an LMM. This model quantifies the relationships between a continuous dependent variable and various predictor variables in a set of longitudinal data, thereby allowing within- and between-subject variations to be incorporated [26]. Therefore, we believe that our results are representative despite the limitations associated with the retrospective nature of our present study. Second, the ankle kinematics parameters measured using 3D gait analysis were considered to be the dependent variables in our study. However, the ankle kinematics assessed did not reflect motion in the coronal or transverse planes. Despite this limitation, we believe that our data are nevertheless very helpful in understanding changes in ankle kinematics after knee surgery.

## Conclusions

We conclude that improvement in total knee ROM is correlated with improvement in ankle kinematics after DHL and DHL with RFT. Knee surgery might influence ankle kinematics during gait to the extent that additional surgical interventions involving the ankle joint become unnecessary. Furthermore, because motion of the knee and ankle joints is cross-linked, surgeons should be aware of potential changes in the ankle joint after knee surgery.

## Additional file

Additional file 1: Univariate model. (DOCX 15 kb)

## Abbreviations

3D: three-dimensional
AIC: Akaike information criterion
BIC: Bayesian information criterion
CI: Confidence intervals
CP: Cerebral palsy
DHL: Distal hamstring lengthening
GMFCS: Gross motor function classification system
LMM: Linear mixed model
RFT: Rectus femoris transfer
ROM: Range of motion
SEMLS: Single-event multilevel surgery
