# Supplementary material for: Influence of surgery involving tendons around the knee joint on ankle motion during gait in patients with cerebral palsy
Source: BMC Musculoskelet Disord. 2018 Mar 15;19:82. doi: 10.1186/s12891-018-2003-0 (PMC5856371; doi:10.1186/s12891-018-2003-0)
Supplement: Supplementary file 1 — Univariate model. (DOCX 15 kb) [file 12891_2018_2003_MOESM1_ESM.docx]

| Supplement. Univariate model. | | | | | | | | | | | | | | | |
| --- | --- | --- | --- | --- | --- | --- | --- | --- | --- | --- | --- | --- | --- | --- | --- |
| Effect | Peak ankle dorsiflexion at IC | | |  | Peak ankle dorsiflexion during stance | | |  | Peak ankle dorsiflexion during swing | | |  | Dynamic ROM of the ankle | | |
|  | Estimate | CI | *P*-value |  | Estimate | CI | *P* -value |  | Estimate | CI | *P* -value |  | Estimate | CI | *P* -value |
| Intercept | 1.7 | -2.3 – 5.7 | 0.394 |  | 16.7 | 12.4 – 20.9 | <0.001 |  | 15.8 | 13.4 – 18.1 | <0.001 |  | 9.1 | 4.3 – 13.9 | <0.001 |
| Sex(Male) | 4.4 | -1.04 – 9.8 | 0.101 |  | 5.8 | -0.02 – 11.6 | 0.051 |  | 0.6 | -2.6 – 3.7 | 0.712 |  | 6.9 | -0.3 – 12.4 | 0.052 |
| Intercept | 2.1 | -1.6 – 5.8 | 0.248 |  | 16.3 | 12.4–20.1 | <0.001 |  | 13.9 | 11.9 – 15.9 | <0.001 |  | 10.8 | 6.3 – 15.3 | <0.001 |
| Minimum knee flexion in the stance | 0.2 | -0.04 – 0.4 | 0.114 |  | 0.3 | -0.01–0.5 | 0.207 |  | 0.2 | -0.1 – 0.3 | 0.120 |  | 0.2 | -0.1 – 0.4 | 0.176 |
| Bold value, *p* < 0.05  CI = 95% confidence interval, IC = initial contact, ROM = range of motion | | | | | | | | | | | | | | | |
